# Activation of mineralocorticoid receptor by ecdysone, an adaptogenic and anabolic ecdysteroid, promotes glomerular injury and proteinuria involving overactive GSK3β pathway signaling

**DOI:** 10.1038/s41598-018-29483-7

**Published:** 2018-08-15

**Authors:** Minglei Lu, Pei Wang, Yan Ge, Lance Dworkin, Andrew Brem, Zhangsuo Liu, Rujun Gong

**Affiliations:** 1 Institute of Nephrology, Blood Purification Center, the First Affiliated Hospital of Zhengzhou University, Zhengzhou, China; 2 Division of Kidney Disease and Hypertension, Department of Medicine, Brown University School of Medicine, Providence, Rhode Island United States; 3 Division of Nephrology, Department of Medicine, University of Toledo College of Medicine, Toledo, Ohio United States

## Abstract

Ecdysone is an arthropod molting hormone and has been marketed as a non-androgenic natural anabolic and adaptogen. However, the safety profile of ecdysone is largely undetermined. After ecdysone treatment for 2 weeks, mice developed albuminuria with histologic signs of glomerular injury, including hypertrophy, mesangial expansion, mild glomerulosclerosis and podocyte injury. A direct glomerulopathic activity of ecdysone seems to contribute, since addition of ecdysone to cultured glomerular cells induced cytopathic changes, including apoptosis, activation of mesangial cells, podocyte shape changes and a decreased expression of podocyte markers. To explore the molecular target responsible for the pathogenic actions, we employed an *in silico* modeling system of compound-protein interaction and identified mineralocorticoid receptor (MR) as one of the top-ranking proteins with putative interactions with ecdysone. The molecular structure of ecdysone was highly homologous to mineralocorticoids, like aldosterone. Moreover, ecdysone was capable of both inducing and activating MR, as evidenced by MR nuclear accumulation in glomerular cells both *in vitro* and *in vivo* following ecdysone treatment. Mechanistically, glycogen synthase kinase (GSK) 3β, which has been recently implicated in pathogenesis of glomerular injury and proteinuria, was hyperactivated in glomeruli in ecdysone-treated mice, concomitant with diverse glomerulopathic changes. In contrast, spironolactone, a selective blockade of MR, largely abolished the cytopathic effect of ecdysone *in vitro* and attenuated albuminuria and glomerular lesions in ecdysone treated mice, associated with a mitigated GSK3β overactivity in glomeruli. Altogether, ecdysone seems able to activate MR and thereby promote glomerular injury and proteinuria involving overactive GSK3β pathway signaling.

## Introduction

Glomerular disease is one of the leading causes of progressive chronic kidney disease and loss of kidney function, culminating in end stage renal disease (ESRD). Proteinuria is an early clinical manifestation and diagnostic sign of glomerular injury^1,2^. In adults undergoing renal biopsy for evaluation of proteinuria, focal segmental glomerulosclerosis (FSGS) accounts for 39% of all cases^3^ and up to 50% of cases in African-American patients^4^. In the past 40 years, the prevalence of FSGS has been continuously rising^3^. The reason for this increasing incidence of FSGS is largely unknown. Despite the improved pathologic diagnosis, a growing occurrence of secondary FSGS may also contribute. Secondary FSGS may develop as part of other disease conditions, such as HIV infection^5^, renal mass reduction^6^, obesity^7^, or following the use of some drugs, including heroin^8^, sirolimus^9^, or bisphosphonates^10^. In addition, male sex steroid hormones like testosterone or androgen seem to amplify compensatory glomerular and tubular growth, which may promote glomerulosclerosis^11^. In humans, males have higher incidence of FSGS compared to females^3^ and typically have a faster progression to ESRD^12^. Male patients with autosomal dominant polycystic kidney disease, IgA nephropathy, membranous nephropathy, or chronic renal disease of unspecified etiology progress more quickly to ESRD^13,14^. Likewise, a number of animal studies show that male mice are more susceptible to the development of glomerular sclerosis after adriamycin injury, renal mass reduction or with aging than their female counterparts, underscoring the detrimental role of male sex hormones^13^. Mechanistically, androgens are known to induce oxidative stress^15^ and upregulate components of the renin angiotensin system^16^. Androgen receptor blockade with flutamide was able to attenuate renal damage in hypertension-induced end-organ damage in animal models^17^. Recently, there are a number of case series that associate proteinuria and FSGS with anabolic androgenic steroid abuse in sportsman and in particular in bodybuilders^18^. However, epidemiologic evidence is still scarce to date and there are no adequate animal model studies on this topic. It remains uncertain whether androgen or increased lean body mass is the exact causative factor for FSGS. This is an important issue, because in addition to androgenic steroids, many other non-androgenic anabolic supplements, like ecdysteroids, are also commonly used for fitness. It is unknown whether these non-androgenic supplements may also pose a risk of impairing kidney health.

Ecdysteroids are one of the most common and major ingredients found in various non-androgenic fitness or adaptogenic supplements^19,20^. They are steroidal insect molting hormones essential for inducing metamorphosis in arthropods, like Drosophila melanogaster, and activate the ecdysone receptor (EcR), a member of the nuclear receptor superfamily^21^. Since EcR is not available in any mammalian cells, it has long been assumed that ecdysteroids may have no effects in mammals. Nevertheless, a wide array of effects have been noted in rodents and humans after using ecdysteroids with a peculiar pro-growth and anabolic activity^22^. To this end, ecdysteroids, represented by ecdysone, have been popularly applied as adaptogenic supplements for improving well-being and in particular used by competitive bodybuilders to achieve impressive muscular physiques^23^. However, the safety profile of ecdysone is largely unknown. This is especially concerning because the doses of ecdysone used for sports or fitness purposes are remarkably high (in a range of up to 1000 mg/day)^19^. Moreover, there are studies associating the use of ecdysone with evident signs of aberrant kidney homeostasis, such as kidney growth and enlargement^24,25^. Therefore, provided that use of anabolic androgenic steroids poses a risk of renal impairment, it is imperative to determine the effect of ecdysone on the kidney health. This study, by using both mice and kidney cell culture models, revealed a mineralocorticoid-like effect of ecdysone that elicits albuminuria and induces early signs of glomerular damage.

## Results

### Ecdysone treatment elicits albuminuria and glomerulopathy in mice

**Figure 1:**
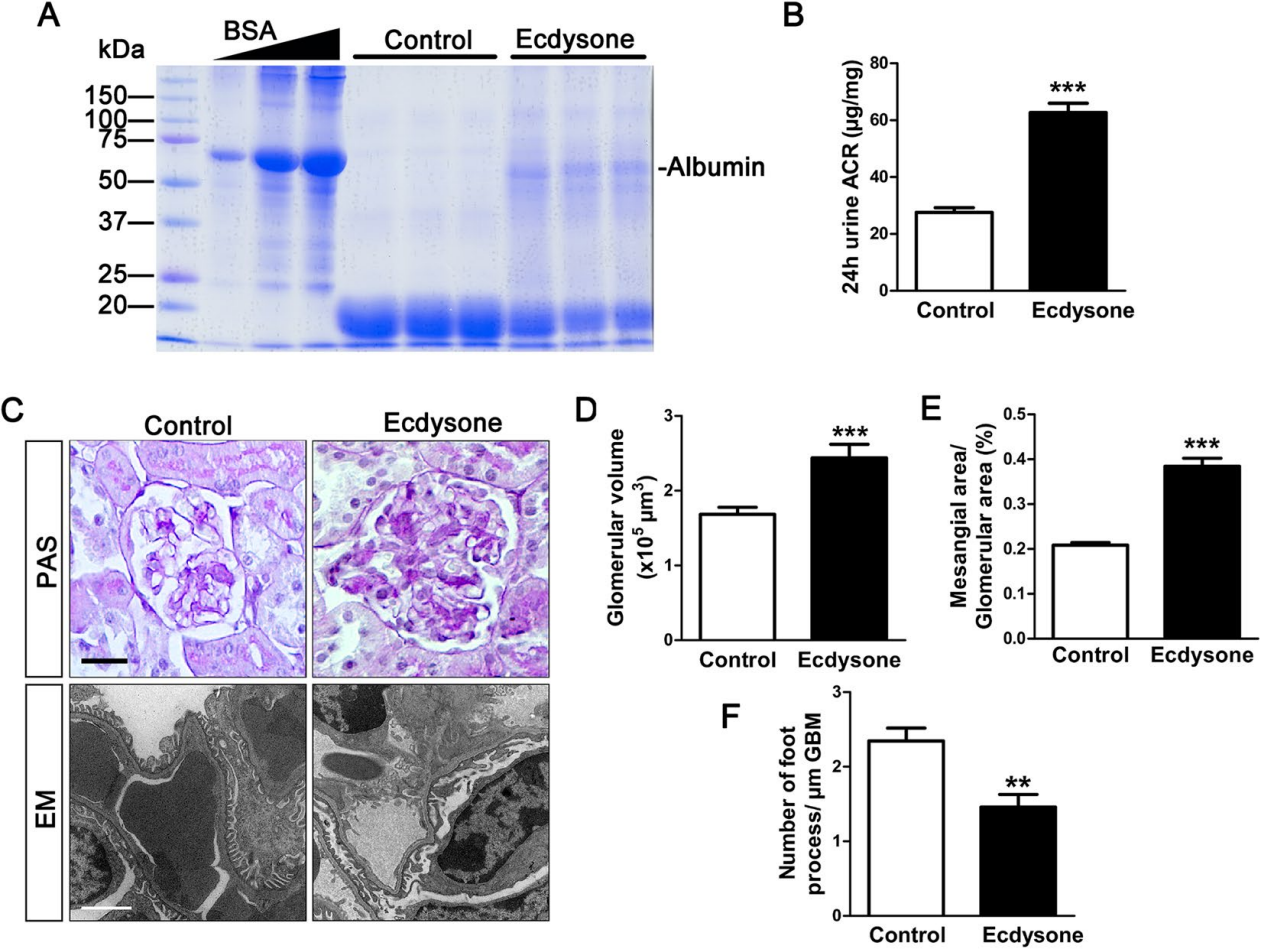
Ecdysone treatment elicits albuminuria and histologic signs of glomerulopathy. Mice received daily subcutaneous injection of ecdysone (6 µg/g/d) or equal volume of solvent vehicle (DMSO) for 2 weeks. (**A**) Urine was collected on day 14 and subjected to SDS-PAGE and staining with Coomassie Brilliant Blue. Bovine serum albumin (BSA) 5, 10, and 20 μg, served as standard control. Urine samples (15 μL) collected from each group were loaded. (**B**) Quantification of urine albumin levels adjusted with urine creatinine concentrations, ***P < 0.001 compared with control group (n = 6). (**C**) Mice were sacrificed on day 14 and kidneys collected and processed for periodic acid–Schiff (PAS) staining for light microscopy (Bar = 20 µm) or for electron microscopy (EM, Bar = 2 µm). (**D**,**E**) Computerized morphometric analysis of glomerular volumes and mesangial-to-glomerular area ratios, ***P < 0.001 *vs* control group (n = 6). (**F**) Absolute count of the number of foot processes per unit length of glomerular basement membrane (GBM) on electron micrographs of kidney specimens. **P < 0.01 *vs* control group *(*n = 6).

Mice received daily subcutaneous treatment with ecdysone or vehicle for two weeks and then euthanized. Before sacrifice, urine was collected and subjected to SDS-PAGE followed by Coomassie Brilliant Blue staining, which showed significant increase in urinary excretion of protein with a molecular weight in the range of 60 kDa that is equivalent to that of albumin (Fig. 1A). This was confirmed by a urine albumin to creatinine ratio, which demonstrated an approximately 2~3 fold increase in albuminuria levels in ecdysone treated mice as compared to vehicle treated mice (Fig. 1B). Kidneys were harvested and processed for Periodic acid–Schiff (PAS) staining, which illustrated evident early signs of glomerulopathy, including mesangial expansion and glomerular enlargement (Fig. 1C). Computerized morphometric analysis quantified the size of glomeruli as well as the area of the glomerular mesangium and confirmed a modest glomerulomegaly (Fig. 1D) and mesangial expansion (Fig. 1E) in mice after ecdysone treatment. Moreover, electron microscopy of kidney specimens revealed a variable degree of podocyte injury ranging from minimal to moderate, marked by focal podocyte foot process effacement (Fig. 1C), which was quantified by absolute count of the number of foot processes per unit length of glomerular basement membrane (Fig. 1F). No changes in systemic hemodynamics as estimated by blood pressure (data not shown) were noted and associated with the ecdysone elicited albuminuria and kidney pathology.

### Ecdysone treatment results in early signs of glomerulosclerosis and podocyte injury in mice

**Figure 2:**
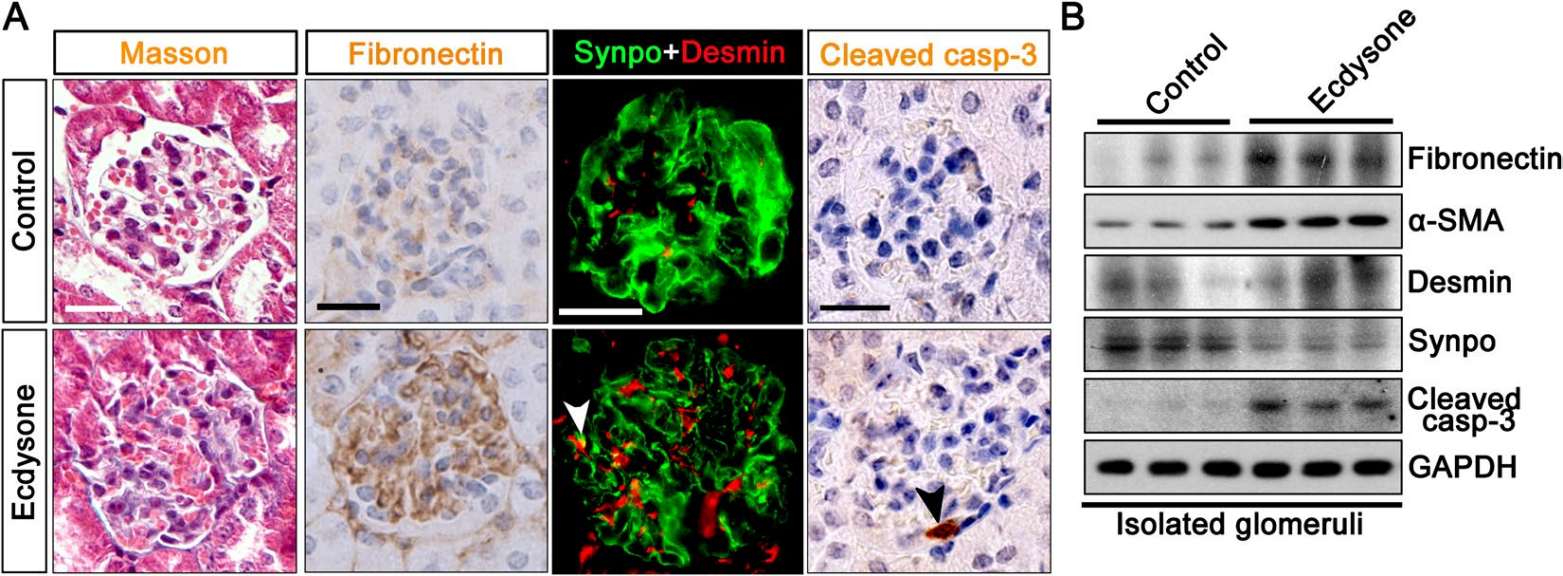
Ecdysone induces glomerular molecular changes indicative of glomerulopathy. (**A**) Mice were treated as stated in Fig. 1. Kidney specimens were obtained on day 14 and prepared for Masson trichrome staining, immunohistochemistry staining for fibronectin and cleaved caspase-3 (Cleaved casp-3), and dual color fluorescent immunohistochemistry staining for synaptopodin (Synpo, green signal) and desmin (red signal). Bars = 20 µm. Ecdysone induced podocyte injury, marked by the reduced expression of synaptopodin and augmented expression of desmin in synaptopodin positive podocytes (yellow signal as indicated by the white arrowhead). Apoptotic cells, probed by positive staining for cleaved caspase-3, were predominantly located to the periphery of glomerular tufts that is consistent with podocyte localization (indicated by the black arrowhead). (**B**) Glomeruli were isolated from kidneys by the magnetic beads-based approach and homogenized for immunoblot analysis for indicated molecules, including fibronectin, α-smooth muscle actin (α-SMA), desmin, synaptopodin (Synpo), cleaved caspase-3 (Cleaved casp-3) and glyceraldehyde 3-phosphate dehydrogenase (GAPDH) (cropped blots are shown as indicated by the lines and full-length blots are included in the Supplementary Figure 1).

To scrutinize the effect of ecdysone on molecular changes in glomeruli, kidney specimens were processed for in-depth histologic examinations and immunoblot analysis. Shown in Fig. 2A, Masson trichrome staining displayed an increase in the amount of blue-colored substance in glomeruli in ecdysone treated animals that corresponds to collagen. Peroxidase immunohistochemistry staining also showed evident mesangial deposition of fibronectin (Fig. 2A), another major constituent of extracellular matrix in glomeruli, underscoring an early sign of glomerulosclerosis. Immunoblot analysis of isolated glomeruli corroborated the morphologic finding on fibronectin expression (Fig. 2B), and revealed that the expression of α-smooth muscle actin, a marker of mesangial cells activation, was significantly up regulated in glomeruli prepared from ecdysone treated animals (Fig. 2B). Desmin, another marker of mesangial activation, was minimally noted in glomeruli from vehicle treated mice, but evidently present in glomeruli after ecdysone treatment. The expression of podocyte specific molecules, like synaptopodin, was substantially diminished in glomeruli in ecdysone treated animals, as shown by both fluorescent immunohistochemistry staining (Fig. 2A) and immunoblot analysis (Fig. 2B). This was associated with podocyte apoptosis, because immunostaining demonstrated an increased expression of cleaved caspase-3, a proteolytic enzyme crucial for the execution of apoptosis, in the kidney from ecdysone treated mice, which was predominantly located to the periphery of glomerular tufts consistent with podocyte localization (Fig. 2A).

### Ecdysone exhibits a direct cytopathic effect on podocytes in culture

**Figure 3:**
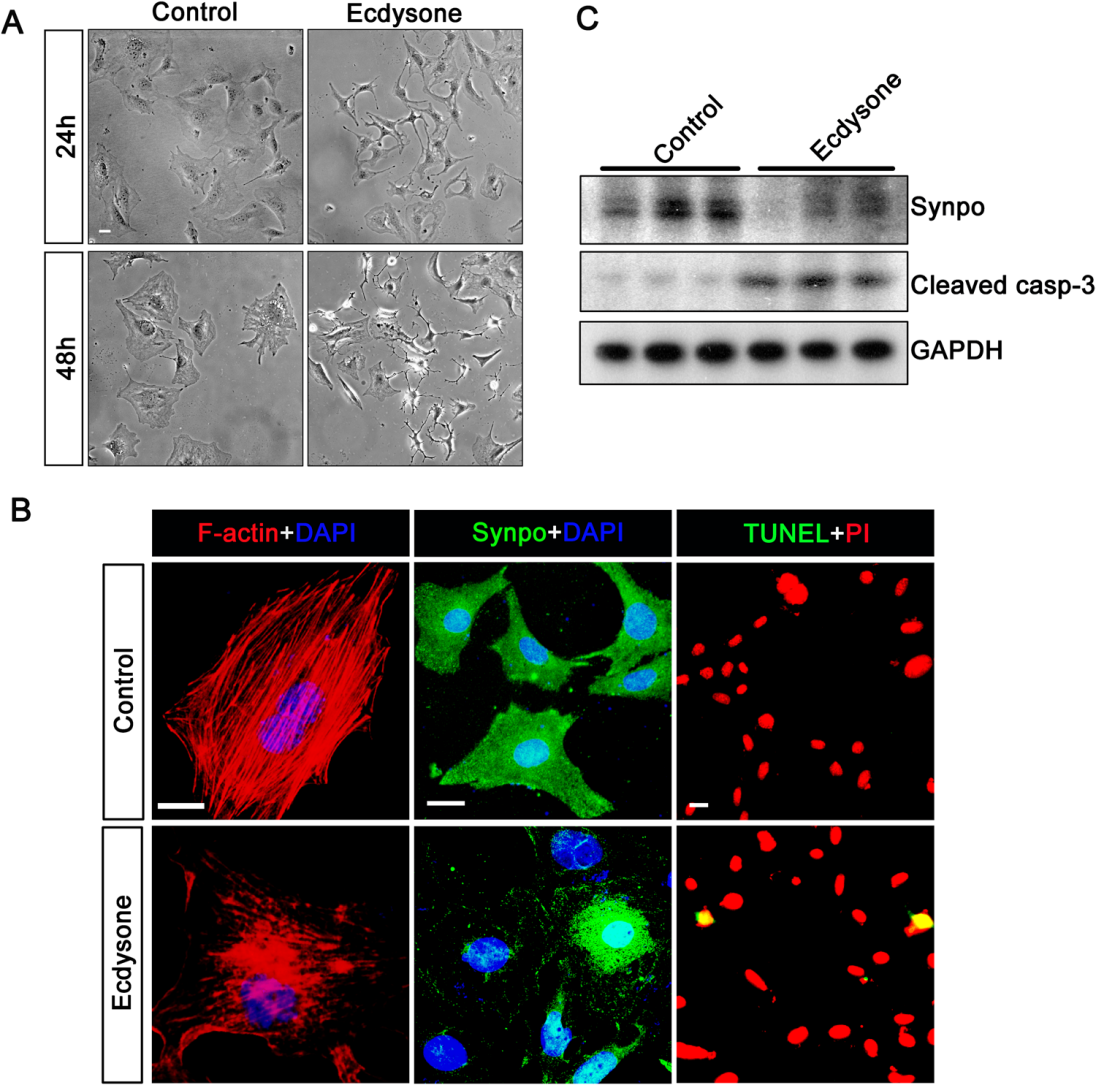
Ecdysone exerts a direct injurious effect on glomerular podocytes. (**A**) Conditionally immortalized mouse podocytes in culture were treated with ecdysone (10^−7^ M) or vehicle for 24 or 48 h. Representative phase contrast micrographs demonstrate podocyte shape changes. Bar = 20 µm. (**B**) Cells were fixed at 48 h and processed for staining for cytoskeletal F-actin with rhodamine phalloidin, terminal deoxynucleotidyl transferase dUTP nick end labeling (TUNEL) staining or for fluorescent immunocytochemistry staining for synaptopodin (Synpo). Cells were counterstained with 4′,6-diamidino-2-phenylindole (DAPI) or propidium iodide (PI). Bars = 20 µm. (**C**) Podocyte cell lysates were collected at 48 h and processed western blot analysis for synaptopodin (Synpo), cleaved caspase-3 (Cleaved casp-3) and glyceraldehyde 3-phosphate dehydrogenase (GAPDH). (cropped blots are shown as indicated by the lines and full-length blots are included in the Supplementary Figure 2).

Under the basal conditions with vehicle treatment, conditionally immortalized mouse podocytes in culture displayed a typical morphology of normal podocytes, featured by large, flat arborized cellular shape with well-developed neurite-like extensions and microvilli. Ecdysone treatment for 24 or 48 h caused cell shrinkage, resulting in an aster-like cell shape (Fig. 3A). The cytologic changes were consistent with signs of podocyte injury, characterized by disruption of the phalloidin-labeled filamentous actin (F-actin) network and diminished expression of synaptopodin, a podocyte specific actin-associated protein, as shown by fluorescent immunocytochemistry staining (Fig. 3B). Moreover, ecdysone elicited apoptosis in cultured podocytes, as detected by TUNEL staining. The morphologic findings were further corroborated by immunblot analysis of cells lysates for synaptopodin and cleaved caspase-3(Fig. 3C). Thus, ecdysone appears to have direct injurious effect on podocytes.

### Ecdysone elicits mesangial cell activation

**Figure 4:**
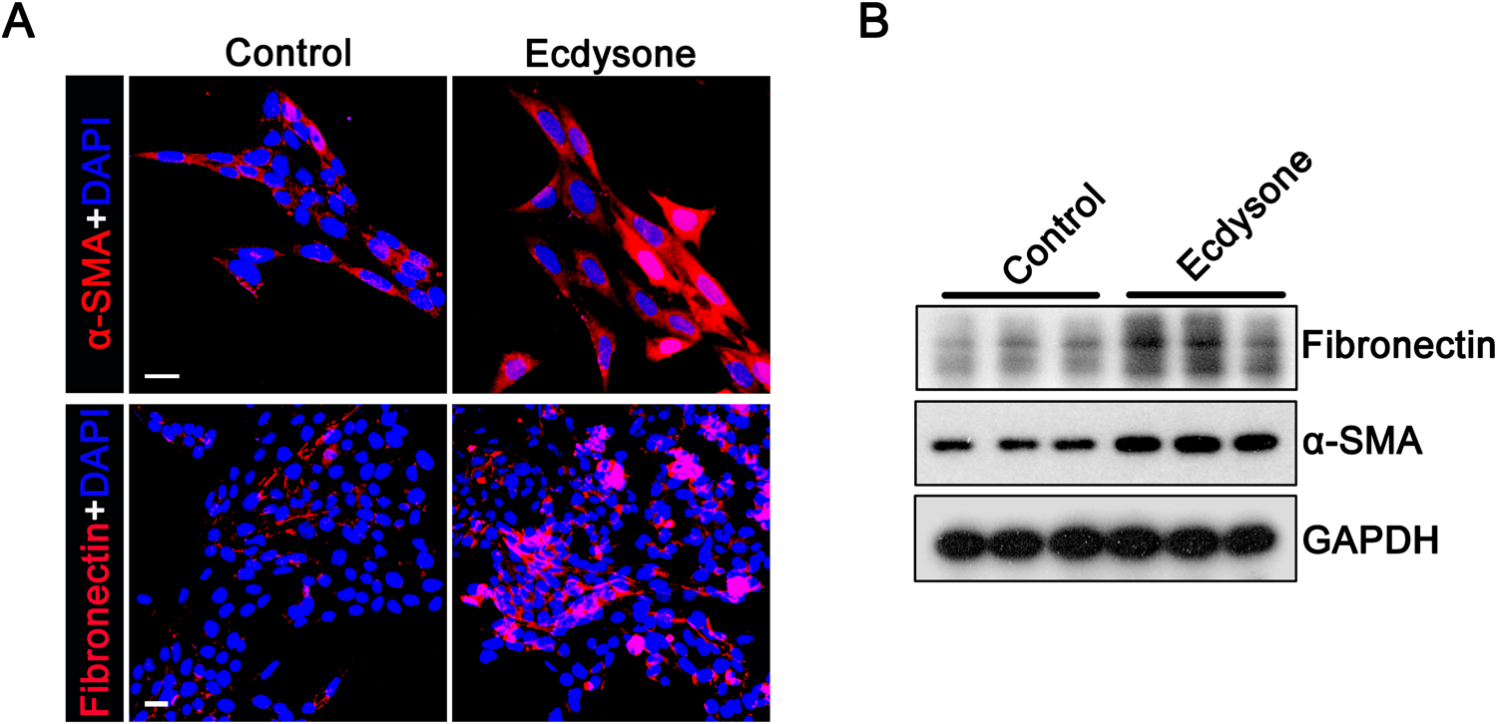
Ecdysone directly induces glomerular mesangial cell activation and extracellular matrix accumulation. (**A**) Mouse mesangial cells were treated with ecdysone (10^−7^ M) or vehicle for 48 hours. Cells were fixed and processed for staining for fluorescent immunocytochemistry staining for α-SMA, a marker of mesangial activation, or the extracellular matrix component fibronectin. Cells were counterstained with 4′,6-diamidino-2-phenylindole (DAPI). Bars = 20 µm. (**B**) Mesangial cell lysates were collected at 48 hours and processed for western blot analysis for α-smooth muscle actin (α-SMA) and glyceraldehyde 3-phosphate dehydrogenase (GAPDH). (cropped blots are shown as indicated by the lines and full-length blots are included in the Supplementary Figure 3).

Since animal studies indicated that ecdysone treatment caused mesangial expansion and glomerulosclerosis, it is conceivable that ecdysone might have an effect on glomerular mesangial cells. To test this hypothesis, cultured mouse mesangial cells were exposed to vehicle or ecdysone for 48 h. Shown in Fig. 4A, ecdysone-treated mesangial cells exhibited activation and contractile activity, marked by a drastic induction of α-SMA, as shown by both fluorescent immunocytochemistry staining (Fig. 4A) and immunoblot analysis of cell lysates (Fig. 4B). In addition, the accumulation of extracellular matrix components like fibronectin was considerably promoted by ecdysone, suggesting a potential profibrotic activity.

### Ecdysone is highly similar to mineralocorticosteroids in molecular structure with MR being a predicted biological target

**Figure 5:**
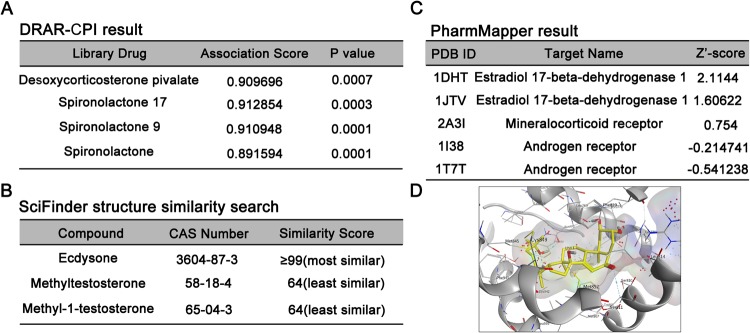
Ecdysone is highly homologous to mineralocorticoids in both molecular structure and biological target. (**A**) Ecdysone was entered as a query molecule into DRAR-CPI, an open-accessed web server for predicting chemical-protein interactions by mining the chemical-protein interactome. Selected high rank compounds similar to ecdysone were shown according to the association scores between ecdysone and the library drugs based on their interaction profiles towards the targets. The association score reflects the magnitude of association between ecdysone and library drugs. *P*-value evaluates the probability of the similarity between the library drug and ecdysone. The smaller the *P*-value is, the greater the association will be. (**B**) The structure of ecdysone is different from testosterones by using the SciFinder website. All the similarity candidate results were grouped by similarity score, and found that testosterones have the least similar to ecdysone. (**C**) Selected high rank drug targets of ecdysone modeled by PharmMapper with Protein Data Bank identification (PDB ID) and Z’-score. (**D**) Mineralocorticoid receptor was identified as putative target protein interacting with ecdysone. MR protein (2A3I, gray color) and ecdysone (yellow color) in the figure are derived from the PDB database. The 3D molecular modeling simulation of compound**–**protein interaction was carried out by MOE method and revealed MR as a molecular target of ecdysone.

To determine putative mechanisms responsible for the effect of ecdysone on glomerular cells, we searched DRAR-CPI (https://cpi.bio-x.cn/drar/), a web server, which calculates the similarities between candidate molecules and available drugs in the Chemical Abstracts Service (CAS) database and predicts biological targets of candidate molecules via chemical-protein interactome. As shown in Fig. 5A, the query of ecdysone with the CAS registry number 3604-87-3 resulted in an answer set of mineralocorticoids, like deoxycorticosterone or mineralocorticoid receptor antagonists like spironolactones, which happen to be similar to the query structure. The association scores between the candidate molecule (ecdysone) and the library drugs were based on their interaction profiles towards the biological targets. However, the structure of ecdysone is likely different from other steroid hormones, like testosterones (Fig. 5B). Chemical-protein interactome predicted that mineralocorticoid receptor (MR), the cognate receptor for diverse mineralocorticoids or mineralocorticoid receptor antagonists, is also a high-likelihood potential biological target for ecdysone (Fig. 5C). In support of this, *in silico* 3-D modeling system and simulations of the protein to ligand interactions demonstrated that ecdysone binds to and perfectly targets the ligand binding domain of MR (Fig. 5D), suggesting that ecdysone might have a direct effect on MR that is akin to mineralocorticoids or mineralocorticoid receptor antagonists.

### Spironolactone counteracts the ecdysone triggered MR activation in glomerular cells *in vitro* and *in vivo*

**Figure 6:**
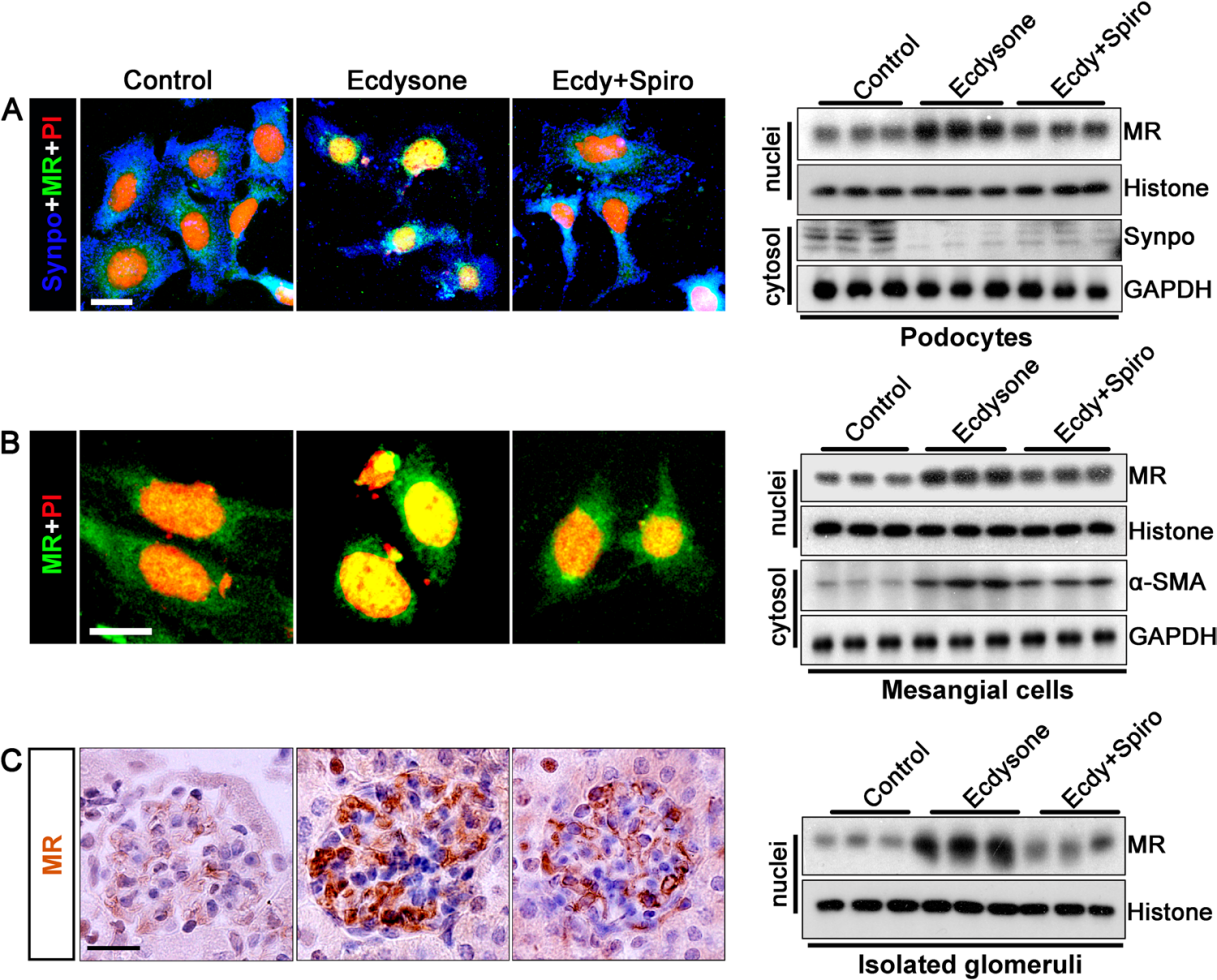
Ecdysone induced mineralocorticoid receptor (MR) nuclear translocation and activation in glomerular podocytes and mesangial cells both *in vivo* and *in vitro*. (**A**) Conditionally immortalized mouse podocytes in culture were treated with ecdysone (Ecdy, 10^−7^ M) or vehicle in the presence or absence of spironolactone (Spiro, 10^−5^ M) for 48 h. Cells were fixed and processed for immunofluorescence staining for MR. Bar = 20 µm. Nuclear fractions and cytoplasmic lysates were processed for western blot analysis for indicated molecules (cropped blots are shown as indicated by the lines and full-length blots are included in the Supplementary Figure 4). (**B**) Cultured mouse mesangial cells were treated with ecdysone (10^−7^ M) or vehicle in the presence or absence of spironolactone (10^−5^ M) for 48 h. Cells were fixed and processed for immunofluorescence staining for MR. Bar = 20 µm. Nuclear fractions and cytoplasmic lysates were processed for western blot analysis for indicated molecules (cropped blots are shown as indicated by the lines and full-length blots are included in the Supplementary Figure 5). (**C**) Mice were treated with ecdysone (6 µg/g/d) or vehicle in the presence or absence of spironolactone (60 µg/g/d). After 2 weeks, mice were sacrificed and kidney specimens processed for peroxidase immunohistochemistry staining for MR. Representative micrographs of glomerular peroxidase staining for MR were shown. Bar = 20 μm. Nuclear fractions were prepared from isolated glomeruli and then subjected to western immunoblot analysis for MR or histone which served as a loading control for protein normalization (cropped blots are shown as indicated by the lines and full-length blots are included in the Supplementary Figure 6). Abbreviations: α-SMA, α-smooth muscle actin; Synpo, synaptopodin; GAPDH, glyceraldehyde 3-phosphate dehydrogenase.

To further determine if ecdysone confers an agonistic or antagonistic effect on MR, cultured podocytes and mesangial cells were processed for fluorescent immunocytochemistry staining for MR and immunoblot analysis. Under basal conditions in vehicle-treated podocytes (Fig. 6A) and mesangial cells (Fig. 6B), MR expression was minimally detected and largely distributed to the cytoplasm. After ecdysone treatment, MR expression markedly increased and significant nuclear translocation of MR could be observed, denoting MR activation. In parallel with MR nuclear accumulation and activation, podocyte injury was seen with loss of synaptopodin in podocytes, and mesangial cell activation, indicated by α-SMA up-regulation in mesangial cells. It seems that MR activation was essential for ecdysone-elicited cytopathic changes, because in the presence of spironolactone, a typical mineralocorticoid receptor antagonist, ecdysone-induced podocyte injury and mesangial cell activation were substantially attenuated, concomitant with a mitigated nuclear import of MR. In consistency, *in vivo* in mice, MR induction and nuclear entry was noted in glomeruli after ecdysone treatment, as revealed by peroxidase immunohistochemistry staining and immunoblot analysis of isolated glomeruli. This effect was blocked in animals co-treated with spironolactone (Fig. 6C).

### MR signaling pathway is responsible for ecdysone-instigated proteinuria and glomerulopathy

**Figure 7:**
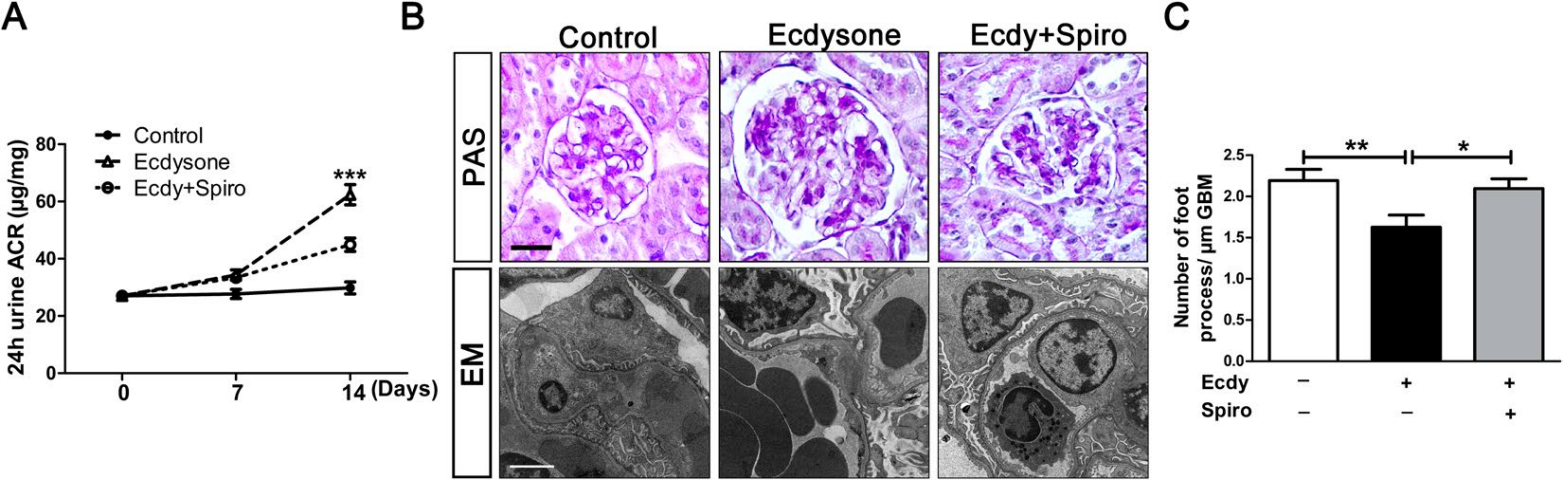
MR signaling pathway is required for the albuminuric and glomerulopathic effects of ecdysone. (**A**) Mice were treated as described in Fig. 6C. On day 0, 7, 14 after various treatments, spot urine was collected and analyzed by mouse albumin ELISA kit and urine creatinine assay kit. Quantification of urine albumin levels adjusted with urine creatinine concentrations; ***P < 0.001 compared with control group and ecdy + spiro group (n = 5). (**B**) Kidney tissues were procured on day 14 and processed for PAS staining for light microscopy (Bar = 20 µm) or for electron microscopy (Bar = 2 µm). (**C**) Absolute count of the number of foot processes per unit length of glomerular basement membrane (GBM) on electron micrographs of kidney specimens. **P < 0.01 *vs* control group, *P < 0.05 *vs* ecdy + spiro group (n = 5).

To determine if MR activation is associated with the harmful effect of ecdysone on the kidney, an additional group of mice were treated with ecdysone in the presence or absence of the spironolactone. Ecdysone-elicited urinary albumin excretion was significantly attenuated in mice co-treated with spironolactone (Fig. 7A). This was in parallel with a decrease in mesangial expansion and extracellular matrix accumulation in glomeruli as shown by PAS staining (Fig. 7B). Moreover, spironolactone blocked the ecdysone induced ultrastructural changes in glomerular podocytes, including the reduced number of podocyte foot processes. (Fig. 7B,C).

### Spironolactone mitigates glomerulosclerosis and podocyte injury elicited by ecdysone in mice

**Figure 8:**
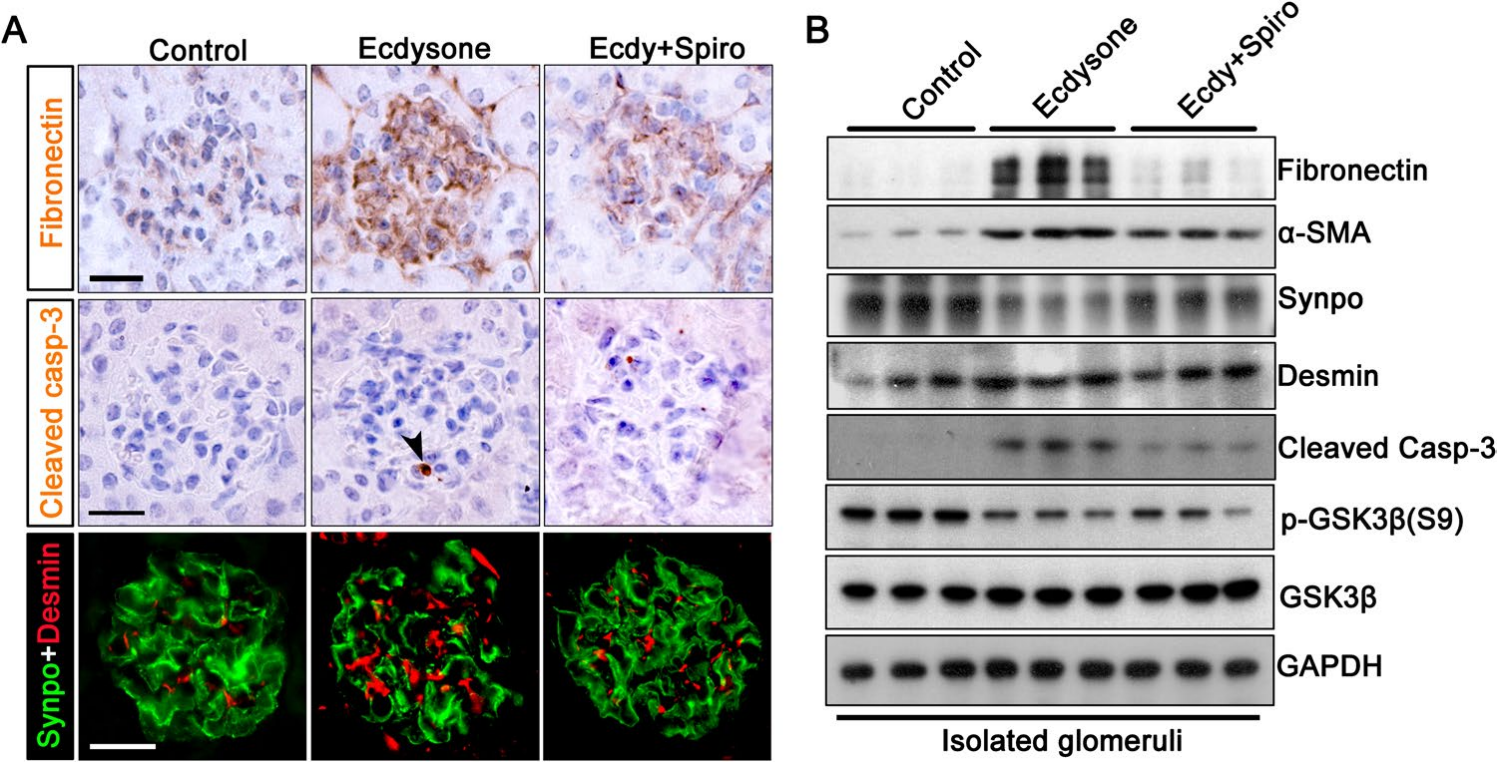
Spironolactone, a selective blockade of MR, mitigates molecular changes in glomeruli in ecdysone treated mice. (**A**) Mice were treated as described in Fig. 6C. Kidney tissues were procured on day 14 and processed for peroxidase immunohistochemistry staining for fibronectin and cleaved caspase-3, or for immunofluorescence staining for desmin (red) and synaptopodin (green). Bars = 20 µm. (**B**) Glomeruli were isolated by the magnetic beads-based approach and prepared for immunoblot analysis for fibronectin, desmin, α-smooth muscle actin desmin (α-SMA), synaptopodin (Synpo), cleaved caspase-3 (Cleaved Casp-3), GSK3β, p-GSK3β (S9) and glyceraldehyde 3-phosphate dehydrogenase (GAPDH). (cropped blots are shown as indicated by the lines and full-length blots are included in the Supplementary Figure 7).

To discern the role of MR in ecdysone induced podocyte injury and early glomerulosclerosis, kidney specimens were processed for peroxidase or fluorescent immunohistochemistry staining. Shown in Fig. 8A, spironolactone significantly attenuated ecdysone-induced glomerular expression of fibronectin, cleaved caspase-3 and desmin, as well as partially restored the expression of the podocyte marker proteins like synaptopodin in ecdysone-treated mice. The morphologic findings were further verified by immunoblot analysis of isolated glomeruli (Fig. 8B). Recently, a growing body of evidence suggests that glomerular injury and proteinuria are associated with, and at least in part, attributable to overactivity of glycogen synthase kinase (GSK)3β pathway signaling in clinical and experimental glomerular diseases^26,27^. In consistency, as shown by immunoblot analysis of isolated glomeruli, ecdysone-triggered albuminuria and early signs of glomerulopathy were concomitant with reduced glomerular expression of GSK3β with inhibitory phosphorylation of at serine 9, indicative of GSK3β hyperactivity. This effect was markedly abolished in animals co-treated with spironolactone. These findings suggest that MR mediates the effect of ecdysone on eliciting podocyte dedifferentiation, glomerular cell apoptosis, mesangial activation and early signs of glomerulosclerosis possibly involving GSK3β overactivity. MR antagonists appear to be able to minimize the harmful activity of ecdysone on glomeruli.

## Discussion

In the present study, we found that ecdysone, an arthropod molting steroid hormone that has been widely used as an anabolic agent or adaptogen, can cause albuminuria. The albuminuria is associated with histologic signs of glomerular injury, including glomerular hypertrophy, mesangial expansion and podocyte injury in mice. Activation of MR is the likely mechanism underlying this glomerulopathic activity. In support of this, *in silico* structure analysis and drug target identification indicated that ecdysone is highly homologous to mineralocorticosteroids in molecular structure with the predicted potential biological target being MR. Moreover, concomitant treatment with spironolactone, a standard MR antagonist, largely prevented the harmful effects of ecdysone. To the best of our knowledge, our study is the first to describe this previously unrecognized renal adverse effect of ecdysone that causes glomerular injury and damages glomerular filtration barrier.

The albuminuria associated with ecdysone appears similar to that induced by anabolic-androgenic steroids like testosterone, which are known to be detrimental to the kidney and cause proteinuria and glomerulosclerosis^28,29^. Although mechanisms at play are unclear, a number of studies point to a direct nephropathic activity of male sex hormones^30^. Indeed, male gender has been known to be a risk factor for poor outcomes of various kidney diseases^13,31^. In addition, male sex hormones, like testosterone, elicit oxidative stress^32^ and activate the renin-angiotensin-aldosterone system^33^, both of which can be detrimental to the kidney. However, this androgen-like harmful effect seems unlikely responsible for ecdysone-elicited kidney damage, because ecdysone is distinct from any male sex hormones in either molecular structure or function. Moreover, androgen receptor is not a likely a biological target for ecdysone^19^. The x-ray structure of the androgen receptor docking with ecdysterone revealed an unfavorable binding pose and a completely different three-dimensional orientation^19^. At those positions where methyl groups are found for the endogenous ligand testosterone, hydroxyl groups sterically interfere with the receptor in case of ecdysterone. In addition, a water molecule mediating important interaction in the testosterone complex is replaced by a methyl group for the ecdysterone docking pose resulting in an unfavorable interaction pattern^19^. In agreement, our data indicated that ecdysone is not similar to testosterone and androgen receptor is unlikely a biological target for ecdysone (Fig. 5). As a matter of fact, ecdysone has been well accepted as a novel class of non-androgenic anabolic agents that exhibited a hypertrophic effect on the fiber size of muscles that was even much stronger than some well-known hormones, like androgenic steroids and insulin growth factor-1^19,34^.

Body mass index (BMI) has been identified recently to be an important risk factor for kidney disease^35,36^. The generalized muscular growth and hypertrophy following ecdysone treatment may increase lean body mass and BMI and lead to a mismatch between body mass and kidney mass. This may result in a scenario of relative low nephron numbers or nephron inadequacy, which requires a compensatory increase in glomerular filtration as a way of renal self-adaptation^37^. In an attempt to meet these demands, individual nephrons will adapt to hyperfiltration through glomerular hypertrophy mediated in part by the renin-angiotensin-aldosterone system^38,39^. As the terminally differentiated cells, podocytes have very limited ability to proliferate during the process of glomerulomegaly and thus are inadequate to cover the expanded glomerular basement membrane. This lack of podocyte coverage is associated with leakage of albumin as well as other proteins through glomerular filtration barrier^40^. Meanwhile, glomerular hyperfiltration and intraglomerular hypertension causes stress-tension, or mechanical stretch, on resident glomerular cells, in particular podocytes and mesangial cells. When the stress persists, extracellular matrix will accumulate in mesangium and podocytes gradually drop out via detachment or apoptosis, eventually leading to segmental scarring of glomerular tufts or postadaptive FSGS^41,42^. The anabolic or hypertrophic effect of ecdysone is not limited to muscles but also applies to organ systems such as the liver and kidney^22,43^. Ecdysone has been demonstrated to directly increase protein synthesis and hypertrophy in a variety of cell types^22,44^. However, kidney hypertrophy is likely a prelude to progressive chronic kidney disease. Indeed, in both human patients and animal models, renal hypertrophy is usually associated with renal injury in a number of pathophysiological conditions such as diabetes, renal mass reduction, hypokalemic nephropathy, chronic metabolic acidosis, high dietary protein intake and others^45^. The mechanism accounting for the direct kidney hypertrophic effect of ecdysone is unclear. But our studies revealed that ecdysone possesses an activity akin to mineralocorticoids, which are well characterized hormones centrally involved in hypertrophic changes in multiple organs including the kidney^46^. It is known that aldosterone is sufficient to induce glomerular hypertrophy and renal fibrotic changes independent of blood pressure^47^. In addition to a direct hypertrophic action on kidney cells^48^, mineralocorticoids like aldosterone, promote inflammation and fibrosis^49–51^ playing a major role in glomerulosclerosis^52,53^. In support of this, MR blockade attenuates or even reverses proteinuria and glomerular injury in models of hypertrophic kidney diseases due to diverse pathophysiological conditions^54,55^. The present studies clearly show that ecdysone induces its effects directly on the kidney. Those detrimental renal effects are mediated by activation of MR resulting in proteinuria and glomerular injury. Treatment with spironolactone substantially attenuated the ecdysone induced albuminuria and glomerular injury, again underscoring a mineralocorticoid-like mechanism. Admittedly, no studies so far have evaluated the safety of ecdysone in human beings. As such, the findings made here need to be validated in human beings by epidemiologic evidence or large scale clinical trials.

In summary, our study demonstrated that ecdysone treatment seems sufficient to cause albuminuria and glomerular injury in murine models. This harmful effect is likely accounted for by a mineralocorticoid-like activity of ecdysone that is able to activate MR in glomerular cells and trigger cytopathic changes, and can be abolished by the MR blockade spironolactone. Our findings suggest that the use of ecdysone, a popular anabolic and adaptogenic agent, may be problematic, at least for kidney health and thus warrants extra precautions.

## Materials and Methods

### Animal experiment design

Animal studies were approved by the Institutional Animal Care and Use Committee, and conform to the US Department of Agriculture regulations and the NIH’s Guide for human care and use of Laboratory Animals. Male C57BL/6 mice at 8~10 weeks of age and weighing 20–22 g were randomly assigned to the following groups: 1) Control group (n = 6): mice received daily subcutaneous injection of solvent vehicle (dimethyl sulfoxide, Sigma-Aldrich, St. Louis, MO, USA); 2) Ecdysone (Sigma-Aldrich, St. Louis, MO, USA) (n = 6): mice received daily subcutaneous injection of ecdysone at a dose of 6 µg/g/d. To test if MR plays a role in mediating the nephropathic effect of ecdysone, a separate group of mice was treated with ecdysone (6 µg/g/d) in the presence (n = 5) or absence (n = 5) of daily subcutaneous injection of spironolactone (Sigma-Aldrich, St. Louis, MO, USA) (60 µg/g/d). Vehicle treated mice served as controls (n = 5). On day 0, 7, 14 after diverse treatments, spot urine was collected. All animals were sacrificed on day 14 and kidneys resected for further examination.

### Urine albumin and creatinine assay

The concentrations of albumin in urine samples were measured by using mouse albumin ELISA quantitation kit (Bethyl Laboratories Inc., Montgomery, TX). Urine creatinine levels were quantified using a QuantiChrom Creatinine Assay Kit (Bioassay Systems, Hayward, CA). Urine albumin excretion was expressed as urine albumin-to-creatinine ratios (µg/mg).

To discern the composition of urine proteins, urine samples were processed for sodium dodecyl sulfate polyacrylamide gel electrophoresis (SDS-PAGE) followed by staining with Coomassie Brilliant Blue (Sigma-Aldrich, St. Louis, MO).

### Isolation of glomeruli

Mice were anesthetized and perfused by infusing the abdominal artery with 5 ml of PBS containing 8 × 10^7^ Dynabeads M-450 (Dynal Biotech ASA, Oslo, Norway). After perfusion, the kidneys were removed and kidney cortices cut into 1-mm^3^ pieces and digested in collagenase A (1 mg/ml, Sigma-Aldrich) at 37 °C for 30 minutes with gentle shaking. The tissue was pressed gently through a 100-μm cell strainer (BD Falcon, Bedford, MA), and glomeruli containing Dynabeads were then gathered using a magnetic particle concentrator. An aliquot (1:1500) of the glomerular isolate was visualized under a microscope to ensure that the sample contained fewer than five tubular fragments per ×200 field. Most isolated glomeruli were decapsulated, which was similar to what had been reported previously^56^. During the procedure, kidney tissues were kept at 4 °C except for the collagenase digestion at 37 °C.

### Renal morphology assessment

Formalin-fixed and paraffin-embedded kidney tissues were prepared in 4 µm sections. For general kidney histology, sections were processed for Periodic acid-Schiff or Masson Trichrome staining to estimate the severity of kidney injury. One observer performed semi-quantitative morphometric analysis in a blinded manner. Alternatively, Image J software (National Institutes of Health, Bethesda, MD) was used for computerized morphometric analysis. Glomerular diameters were measured in 50 ± 5 gloms/group at 400× magnification. Glomerular volume was determined as *Gv* = *β/κ.(π.r*^2^)^*3/2*^, where β = 1.38 is the shape coefficient for spheres, κ = 1.1 is the size distribution coefficient, and (π.r^2^) is the glomerular area^57^. 50 ± 5 random glomeruli from the kidney section of each animal were sampled for the measurement of relative mesangial areas, which were defined as fraction of mesangial area over glomerular area by using Image J software.

### Electron microscopy

Kidney cortex samples were fixed in 2.5% glutaraldehyde. Then ultrathin sections were cut and mounted on a copper grid. Images were photographed using an EM-10 microscope (Zeiss) operated at 80 kV. For counting the number of podocyte foot processes, 10 ± 5 random electron microscopic fields of glomeruli per animal group were examined. The morphologic features were assessed by a single observer in a blinded manner.

### Cell culture and treatments

Conditionally immortalized mouse podocytes were cultured as previously described^58^. Podocytes were routinely grown on plates coated with collagen I at 33 °C in the presence of interferon γ, and grown at 37 °C in the absence of interferon γ for 14 days to induce differentiation. Mouse glomerular mesangial cells were purchased from ATCC (CRL-1927™) and cultured in DMEM/F12 media containing with 5% Fetal Bovine Serum (FBS) in 5% CO_2_ at 37 °C. For experiments, all cells were seeded on plates until 60–70% confluent, and starved in serum-free medium for 12 hours, then incubated in serum-free medium containing vehicle, Ecdysone (10^−7^ M) or Spironolactone (10^−5^ M) for 24 or 48 hours. All *in vitro* studies were repeated five to six times.

### Cell apoptosis analysis

Cultured cells were fixed with 4% paraformaldehyde in phosphate-buffered saline and processed for staining with a TdT-mediated dUTP nick end labeling (TUNEL) kit (Promega, Madison, WI, USA). TUNEL positive cells were visualized by using the fluorescence microscope (BX43, Olympus, Tokyo, Japan).

### Immunofluorescent staining

Cultured cells or frozen kidney cryostat sections were fixed and processed for fluorescent staining. Samples were stained with primary antibodies against synaptopodin (PROGEN Biotechnik GmbH, Heidelberg, Germany), desmin (1:100, Santa Cruz Biotechnology, CA), MR (1:100, curtesy of Dr. Gomez-Sanchez, University of Mississippi), α-SMA (1:100, Abcam, San Francisco, CA, USA) or fibronectin (1:100, Abcam, San Francisco, CA, USA) overnight at 4 °C, followed by Alexa Fluor-conjugated secondary antibodies (Invitrogen). As a negative control, primary antibodies were replaced by preimmune serum. F-actin in podocytes was stained by rhodamine phalloidin (Molecular Probes, OR, USA). Finally, samples were counterstained with propidium iodide or 4′,6-diamidino-2-phenylindole, mounted with Vectashield mounting medium (Vector Laboratories, Burlingame, CA, USA) and visualized by fluorescence microscope (BX43, Olympus, Tokyo,Japan). ImageJ software was used for post processing of the images (scaling, merging, and colocalization analysis).

### Peroxidase immunohistochemistry analysis

Paraffin-embedded kidneys were prepared into 4 µm thick sections, deparaffinized and rehydrated. For peroxidase immunohistochemistry staining, sections were prepared for heat-induced epitope retrieval by microwave. Immunostaining was performed using primary antibodies against the following molecules, including fibronectin (1:100, Abcam), cleaved caspase-3 (1:100, Cell Signaling Technology, MA, USA), MR (1:100). As a negative control, the primary antibody was replaced by nonimmune serum from the same species.

### Western Immunoblot Analysis

Cultured cells were lysed and kidney tissues were homogenized in radioimmunoprecipitation assay buffer containing protease inhibitors. Total cell lysates or subcellular protein fractionations were prepared and processed for immunoprecipitation or immunoblot analysis as described previously^59^. Antibodies against the following molecules were used as primary antibodies to probe blots: against synaptopodin (1:50, PROGEN Biotechnik GmbH, Heidelberg, Germany), desmin (1:100, Santa Cruz Biotechnology, CA), MR (1:100, curtesy of Dr. Gomez-Sanchez, University of Mississippi), cleaved caspase-3 (1:1000, Cell Signaling Technology, MA, USA), GSK3β and phosphorylated GSK3β (1:1000, Cell Signaling Technology), fibronectin (1:2000, Abcam, San Francisco, CA, USA), α-SMA (1:1000, Abcam, San Francisco, CA, USA), Histone (1:500, Santa Cruz Biotechnology, Santa Cruz, CA), and GAPDH (1:5000, Santa Cruz Biotechnology, Santa Cruz, CA). For immunoblot analysis, the integrated pixel density of bands was determined using the Image J software.

### *In silico* chemical similarity search and biological target identification

DRAR-CPI is an open-accessed web server (http://cpi.bio-x.cn/drar/)^60^ for predicting chemical-protein interactions by mining the chemical-protein interactome. The server gives the positive or negative association scores between a query molecule (ecdysone) and the library drugs based on their interaction profiles towards the targets. According to the association score, library drugs sharing similarity interaction profiles with ecdysone were screened (desoxycorticosterone pivalate and spironolactone).

Draw automatically the structure of ecdysone into SciFinder website (https://origin-scifinder.cas.org/scifinder/) by inputting the CAS Registry Number 3604-87-3, then select the similarity search, all the similarity candidate results were grouped by similarity score. The answers are sorted by similarity score in descending order, finally selected the known chemicals.

For *in silico* drug target predictions we used PharmMapper (http://lilab.ecust.edu.cn/pharmmapper/)^61^ to identify pharmacophore matches common to ecdysone. The 3D molecular modeling simulation study was performed with MOE (The Molecular Operating Environment) Version 2015.10 as described before^62^.

### Statistical analyses

All results are expressed as the means ± SEM. All of the statistical analyses were performed using SPSS 17.0 software. For statistical comparison between two groups, the independent-samples *t* tests were used. For comparison between three groups, one-way ANOVA followed by LSD test was used. *P* values < 0.05 were considered statistically significant.

## Electronic supplementary material

supplementary data
