# Supplementary material for: Activation of mineralocorticoid receptor by ecdysone, an adaptogenic and anabolic ecdysteroid, promotes glomerular injury and proteinuria involving overactive GSK3β pathway signaling
Source: Sci Rep. 2018 Aug 15;8:12225. doi: 10.1038/s41598-018-29483-7 (PMC6093907; doi:10.1038/s41598-018-29483-7)
Supplement: Supplementary file 1 — supplementary data [file 41598_2018_29483_MOESM1_ESM.pdf]

<u>Supplementary Information:</u>

Activation of mineralocorticoid receptor by ecdysone, an adaptogenic and anabolic ecdysteroid, promotes glomerular injury and proteinuria involving overactive GSK3β pathway signaling

Minglei Lu[1,2,3], Pei Wang[1,2], Yan Ge[2], Lance Dworkin[3], Andrew Brem[2], Zhangsuo Liu[1], Rujun Gong[1,2,3]

[1]Institute of Nephrology, Blood Purification Center, the First Affiliated Hospital of Zhengzhou University, Zhengzhou, China; [2]Division of Kidney Disease and Hypertension, Department of Medicine, Brown University School of Medicine, Providence, Rhode Island; [3]Division of Nephrology, Department of Medicine, University of Toledo College of Medicine.

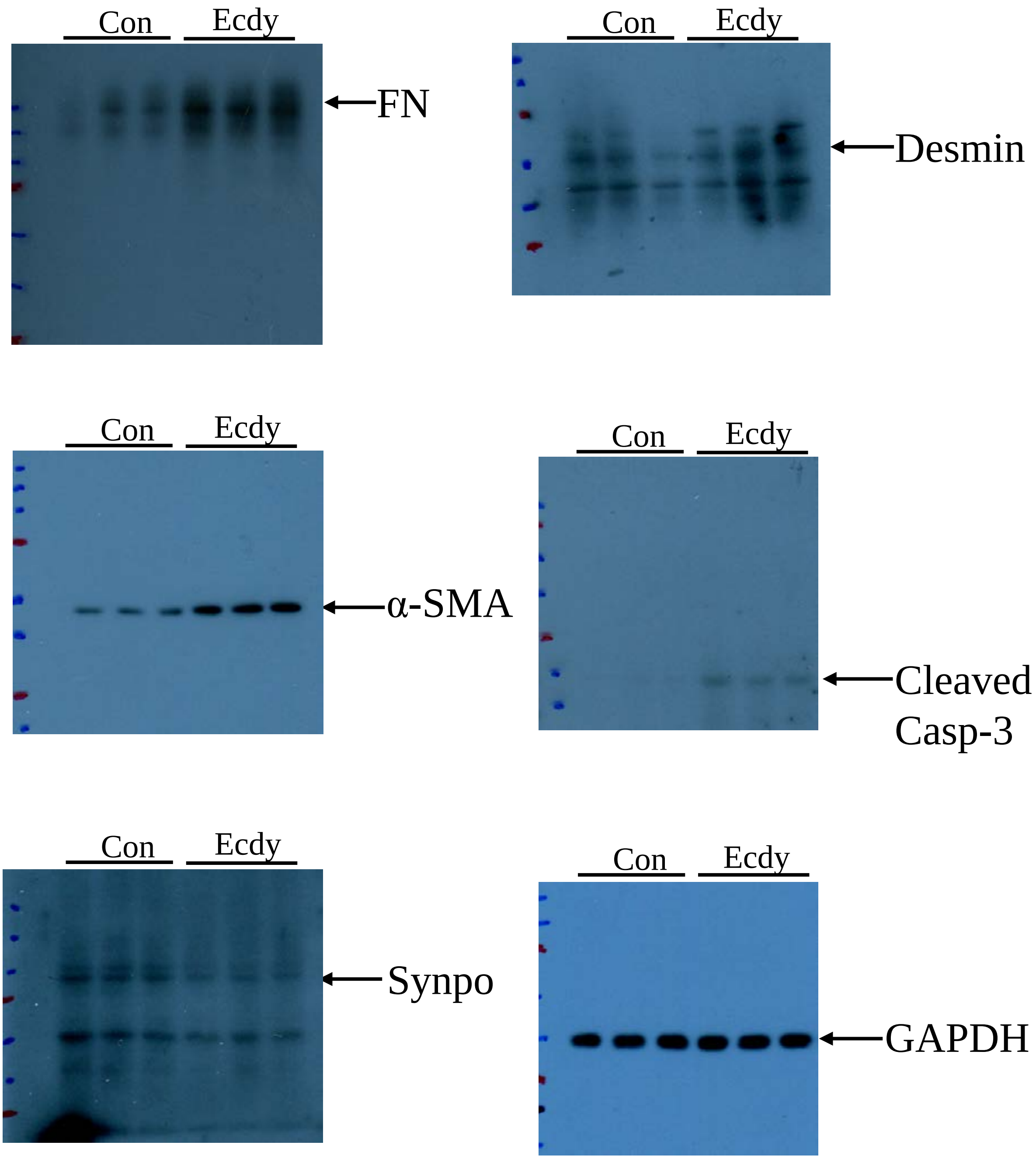

Supplementary Figure 1:Uncropped blots used for the construction of composite of Figure 2.

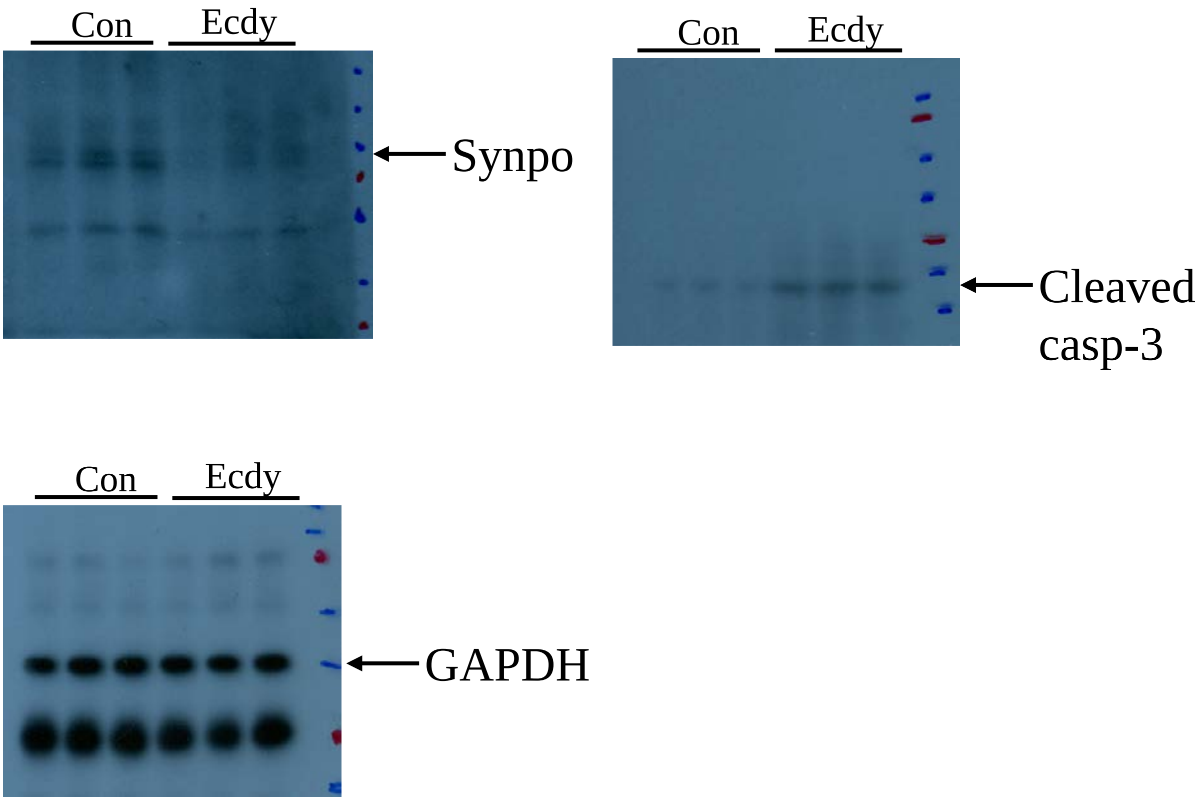

Supplementary Figure 2:Uncropped blots used for the construction of composite of Figure 3.

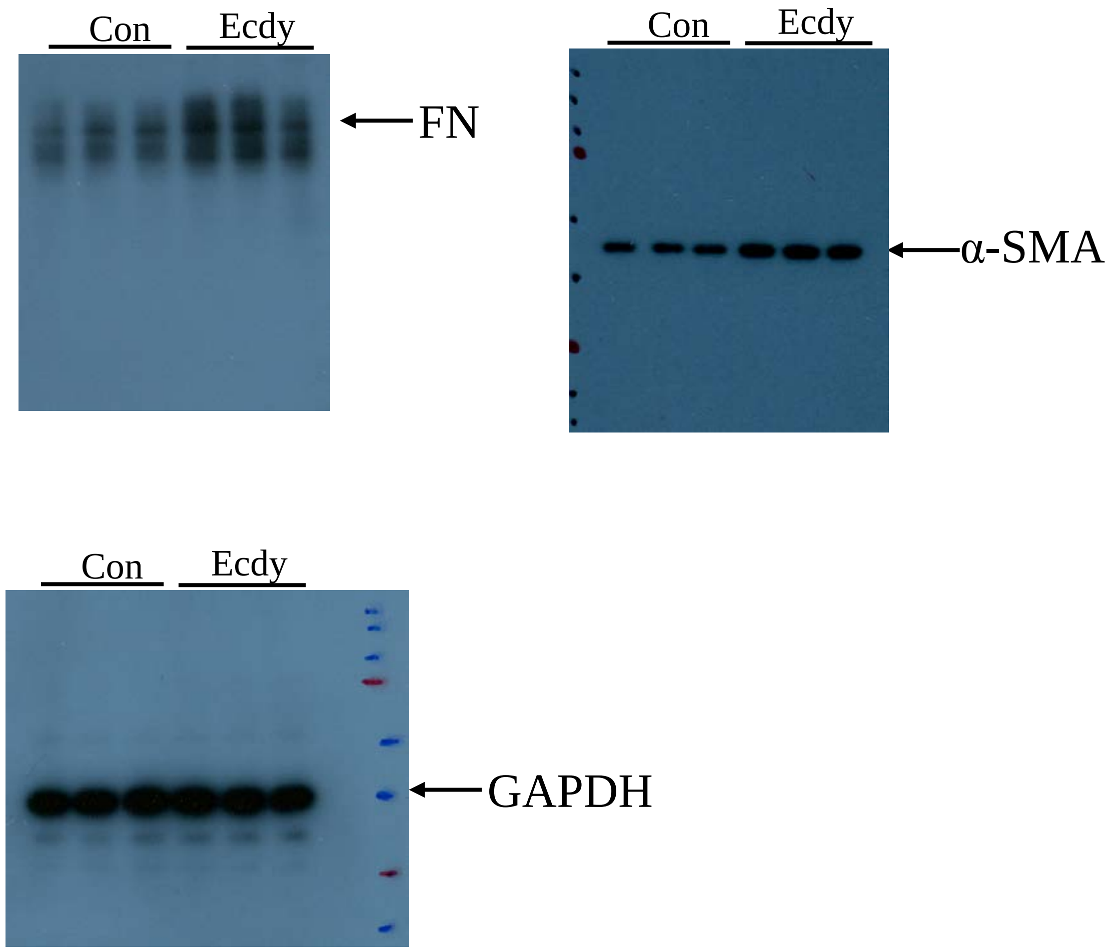

Supplementary Figure 3:Uncropped blots used for the construction of composite of Figure 4.

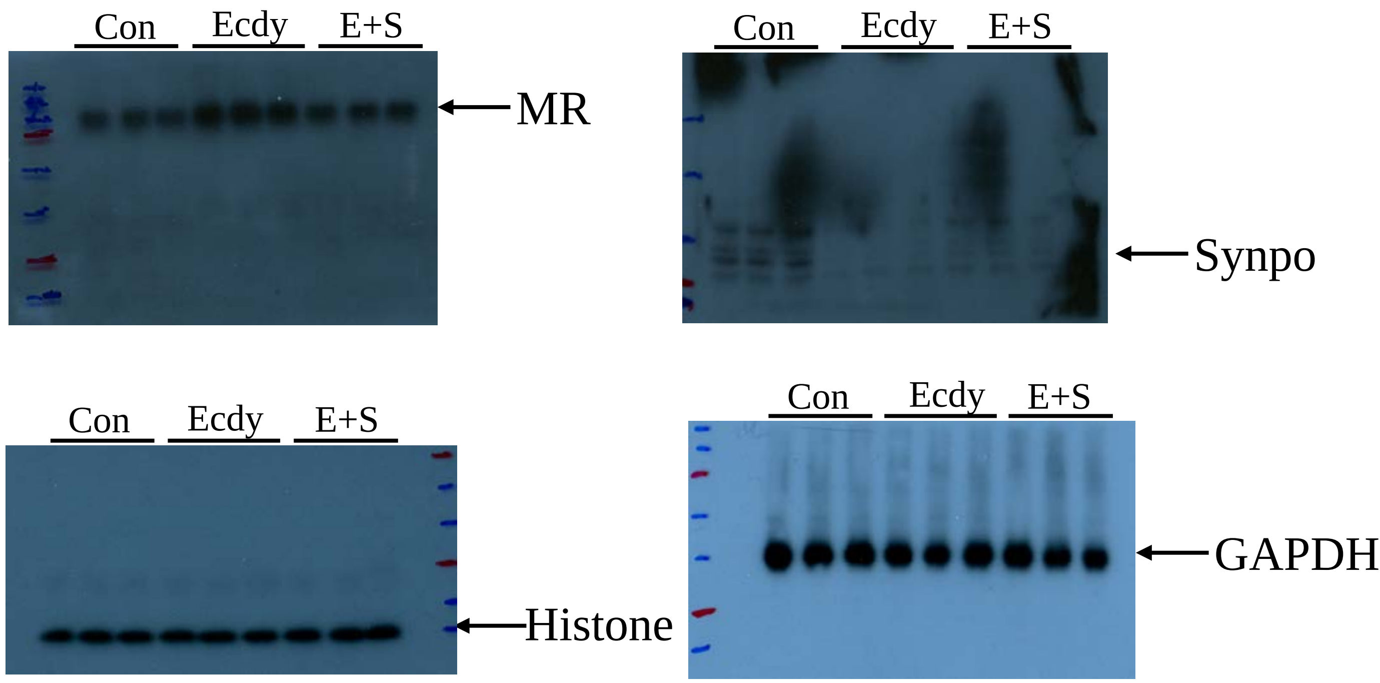

Supplementary Figure 4:Uncropped blots used for the construction of composite of Figure 6A.

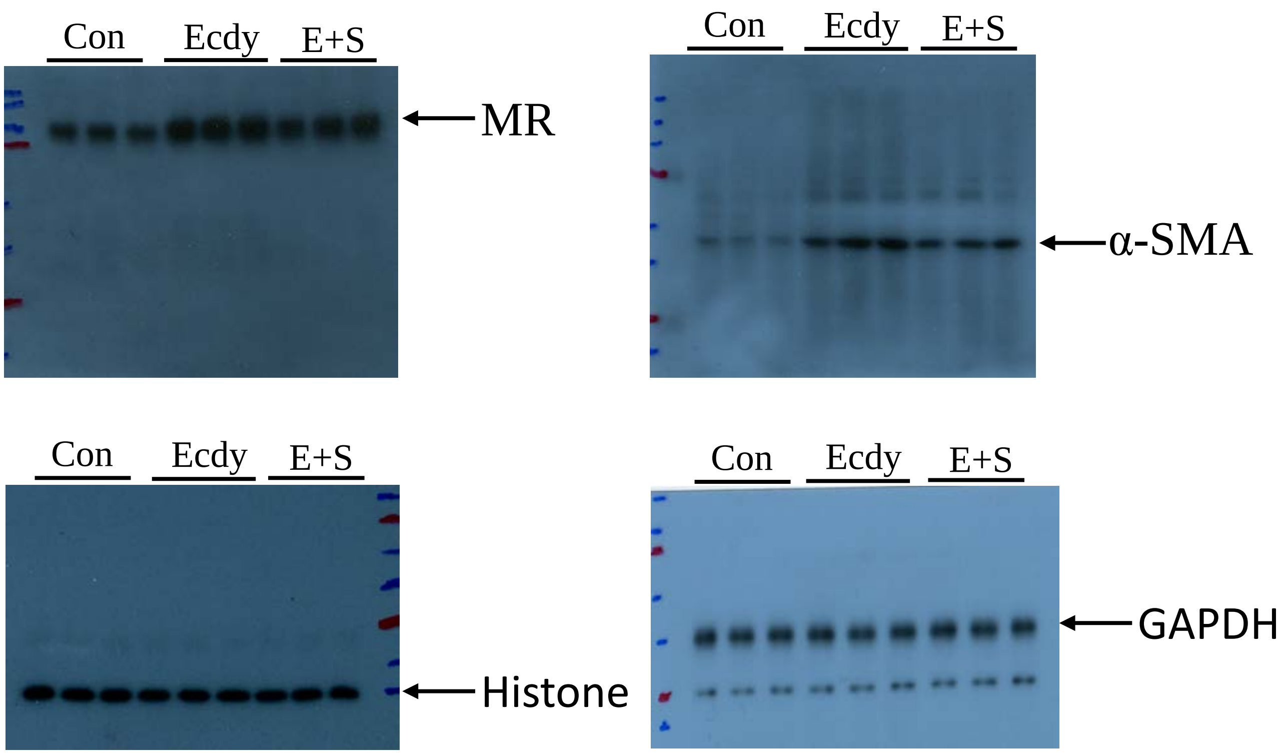

Supplementary Figure 5:Uncropped blots used for the construction of composite of Figure 6B.

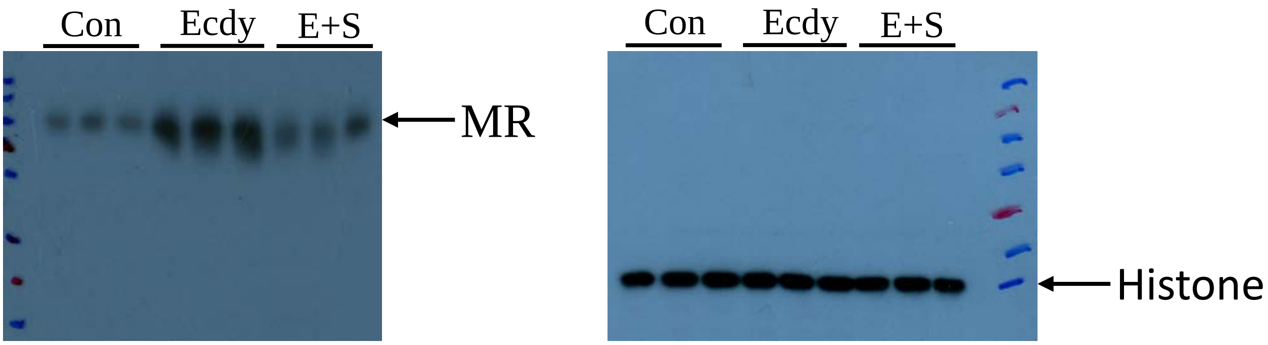

Supplementary Figure 6:Uncropped blots used for the construction of composite of Figure 6C.

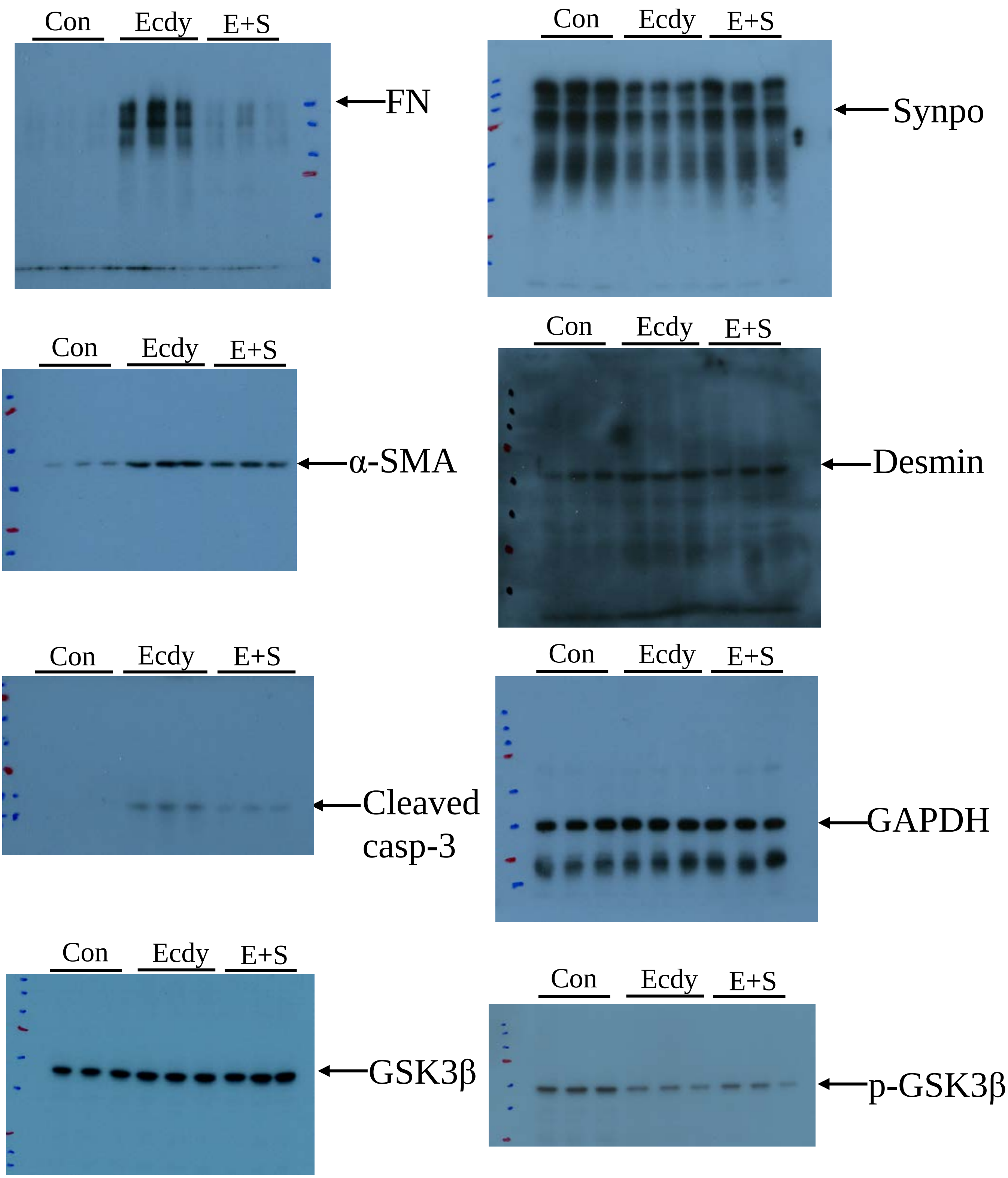

Supplementary Figure 7:Uncropped blots used for the construction of composite of Figure 8.
